# Investigation into the Influence of Physician for Treatment Based on Syndrome Differentiation

**DOI:** 10.1155/2013/587234

**Published:** 2013-10-28

**Authors:** Lijie Jiang, Baoyan Liu, Qi Xie, Shuhong Yang, Liyun He, Runshun Zhang, Shiyan Yan, Xuezhong Zhou, Jia Liu

**Affiliations:** ^1^Institute of Basic Research in Clinical Medicine, China Academy of Chinese Medical Sciences, No. 16 NanXiaoJie, DongZhiMenNei, Dong Cheng District, Beijing 100700, China; ^2^China Academy of Chinese Medical Sciences, No. 16 NanXiaoJie, DongZhiMenNei, Dong Cheng District, Beijing 100700, China; ^3^Community Health Management Center of Dong Cheng District, No. 2 He Ping Li Dong Jie & Jiao Lin Jia Dao Jia, Dong Cheng District, Beijing 100013, China; ^4^Guang'anmen Hospital, China Academy of Chinese Medical Sciences, No. 5 BeiXianGe, GuangAnMenNei, XiCheng District, Beijing 100053, China; ^5^School of Computer and Information Technology, Beijing Jiaotong University, No. 3 Shangyuancun, HaiDian District, Beijing 100044, China

## Abstract

*Background*. The characteristics of treatment based on syndrome differentiation (TBSD) cause great challenges to evaluate the effectiveness of the clinical methods. *Objectives*. This paper aims to evaluate the influence of physician to personalized medicine in the process of TBSD. *Methods*. We performed a randomized, triple-blind trial involving patients of primary insomnia treated by 3 physicians individually and independently. The patients (*n* = 30) were randomly assigned to receive treatments by the 3 physicians for every visit. However, they always received the treatment, respectively, prescribed by the physician at the first visit. The primary outcome was evaluated, respectively, by the Pittsburgh Sleep Quality Index (PSQI) and the TCM symptoms measuring scale. The clinical practices of the physicians were recorded at every visit including diagnostic information, syndrome differentiation, treating principles, and prescriptions. *Results*. All patients in the 3 groups (30 patients) showed significant improvements (>66%) according to the PSQI and TCM symptoms measuring scale. *Conclusion*. The results indicate that although with comparable effectiveness, there exist significant differences in syndrome differentiation, the treating principles, and the prescriptions of the approaches used by the 3 physicians. This means that the physician should be considered as an important factor for individualized medicine and the related TCM clinical research.

## 1. Introduction

Traditional Chinese medicine (TCM) is one of the major schools of complementary and alternative medicine, which has unique theory and abundant preventative and therapeutic methods [1]. The basic features of TCM are holism and treatment based on syndrome differentiation [2]. In the development process, TCM diagnosis and treatment system forms two systems: disease differentiation and syndrome differentiation [3]. Recent years, the mode of combining diseases with syndrome differentiation has become the main way to implement the TBSD [4]. For some simple disease, the TBSD is relatively easy to conduct. However, the processes of most diseases are very complicated, the pathological changes of each stage are different, and accordingly the treatment is mutative. Thus, the treatment based on syndrome differentiation is more preferable. In fact, the clinical treatment is usually made on the basis of disease differentiation and combining the syndrome differentiation [5].

**Figure 1:**
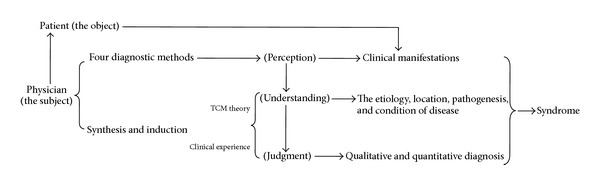
The formation process of the syndrome.

In clinical practice, the treatments are individualized according to the TCM syndrome diagnosis. TCM syndrome, in terms of TCM theory, is defined as a diagnostic classification of the pathological changes of a disease state based on an individual's symptoms and signs, including pulse form and tongue appearance [6]. The formation process of TCM syndrome is as follows (Figure 1): under the guidance of the TCM theory and clinical experience, the physician observes the patient carefully with the four TCM diagnostic methods (inspection, listening and smelling examination, inquiry, and pulse taking and palpation) and grasps the characteristics of clinical symptoms and signs induced by disease. Based on the perception, the physician decides on the cause of the disease according to the rule of “finding the cause with syndrome differentiation” and conducts an analysis considering the environment, time, climate, constitution, gender, history of past illness, and personal history of the patient, so as to identify the essence, location, pathogenesis, and state of the disease and to make the qualitative and quantitative judgment of the clinical manifestation and then determine the TCM syndrome as well as the treatment. Every time the patient sees the physician, the physician needs to adjust the diagnosis, reevaluate the patient's condition, see how the syndrome has changed, and evaluate the therapeutic effect, so as to make a further treatment plan.

Traditional Chinese therapeutics is based on regulating the function and condition of the human body, and TCM syndrome is the summary and description of the state and the way of human motion in a therapeutic perspective [7]. TCM diagnosis and treatment process includes two key entities, namely, disease object (patient) and subject (physician). So the treatment based on syndrome differentiation is an interactive process of physician and patient. Nevertheless, physician plays a leading role in this process [8]. Hence, TBSD is kind of individualized; dynamic; complicated; and sequential diagnosis, treatment, and evaluation process.

TBSD is a typical method of individualized diagnosis and treatment [9, 10]. It means the emphasis of individual characteristics in clinical diagnosis and treatment. The idea of individualized diagnosis and treatment can be traced more than 2000 years ago in the book of Yellow Emperor's Classic of Internal Medicine, which lays a solid theoretical basis. It highlights the principle of treatment according to the climatic and seasonal conditions, geographical localities, and patient's individualities under the guidance of holism [11]. Treatise on Cold Pathogenic and Miscellaneous Diseases (ShangHanZaBingLun) at the end of the east Han dynasty fully embodies the characteristics of individualized diagnosis and treatment by means of its treatment based on syndrome differentiation system. Each formula corresponding to syndrome has main syndrome pattern, which reflect the core characteristic symptoms and signs, which would be the indications for formula using [12]. Furthermore, the syndrome differentiation of Six Meridians reflects the commonality of occurrence and development of diseases; syndrome differentiation of Zang-Fu reveals the individuality of occurrence and development of diseases [13]. Perhaps these theories provide cues and basis or evoke disputes for the evaluation method research.

Due to its unique theory and vast applications in medical practice, evaluation of TCM clinical therapeutic effect has gradually become a hot topic around the world. The office of alternative medicine (OAM) in the United States presents that the efficacy evaluation of traditional/alternative medicine therapies is a critical question in the methodology research report of alternative medicine [14]. Although randomized controlled trial (RCT) is recommended as an international research framework, its application in TCM clinical research is limited [15, 16]. In order to pursue the repeatability, standard, and high quality of the evaluation method, many studies have ignored the characteristics of TBSD and only considered fixed clinical herb prescriptions in clinical studies. For example, the interventions adopted the fixed-prescription or Chinese patent drug [17, 18] or treated the patients of the same syndrome diagnosis as the homogenous group with the same prescription in the whole study period [19–21]. Although many RCT studies have been carried out, high quality research reports accepted by academia are rare; nonrandomized controlled trials are the main methods applied in TCM clinical research [22, 23].

As the current evaluation methods cannot properly reflect the TCM characteristics and advantages, thus, the discussion and strategies on research methods of TBSD are much more concerned [24–28]. Several study design methods, such as pragmatic randomized controlled trials [29], prospective cohort study [30], and single case randomized controlled trial [31], might be more suitable for individualized studies than the classic RCT design [32]. It is considered that the TCM syndrome classification and complex interventions are the core issues which influenced clinical efficacy evaluation [33]; the feature of TBSD requires the therapeutic effect evaluation covering the individualization characteristics and its related factors [34]. Furthermore, as a well-recognized complexity science [35], TCM acts as complex system [36–39] with complicated factors/entities interacting to each other. However, there is little research to investigate and evaluate the factors which influence individualized medicine in TBSD. As one of the important entities involved in TBSD, TCM physician is the subject who manages the whole clinical tasks, which would be expected as a main factor contributing to individualized medicine. Therefore, the objective of this paper is to present a pilot clinical study to investigate the influence of physician for individualized medicine in TBSD, including clinical observation, diagnosis, and treatment.

## 2. The Rationale of the Physicians' Individualities in the Treatment Based on Syndrome Differentiation

### 2.1. The Individualities of the Physicians in the Process of TCM Diagnosis and Treatment

The human body is a complex system, so the same state of life often presents a variety of external manifestation. Due to the differences of TCM theory and clinical experience, the special emphasis of the physicians is also different for the same patient. Usually the primary symptoms, secondary symptoms, the judgment of the tongue manifestation, and the pulse condition are different. For instance, the physicians of Shang Han School collect the symptoms and signs from the point of transmission in Six Meridians; the physicians of Nourishing Yin School pay much more attention to the fluid condition for gain or loss; the physicians of the Nourishing Spleen School are more concerned about the functional abnormalities of the spleen and stomach. In addition, the different experience of the physicians leads to their different understanding and judgment of a certain sign and symptom. For instance, in the judgment of the rapid pulse, the experienced physician can differentiate the rapid and excessive pulse of the high fever patient from the rapid but feeble pulse of the heart failure patient. Moreover, in the judgment of the patient's fatigue in TCM [40], the experienced physician divides it into fatigue, intolerance labor, physical and mental exhaustion, and skills incapability of four types, which, respectively, reflects the function status of spleen, liver, heart, and kidney. Correspondingly, the conditions of the disease become mild to serious.

Thus, physicians observe things from different perspectives, accordingly the perception and understanding are different, which lead to the thinking difference of syndrome differentiation. For signs and symptoms of the patient, the physician can understand them from the site of occurrence, the nature, the causative factor, aggravation factor, and relieving factors. Therefore, the generation of TCM individualized treatment not only depends on the patient's individualities, but also depends more on the physicians TCM theory that they applied and clinical experience that they had.

### 2.2. The Representation of the Individualities of the Physicians in the Process of TCM Diagnosis and Treatment

**Figure 2:**
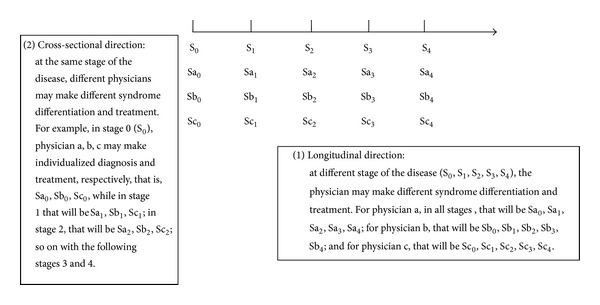
The characteristics of TCM individualized diagnosis and treatment.

For a given patient, there are several stages in the whole process of the disease. Because there are different syndromes in different stages, so the prescription should be changed accordingly. Even if at the same stage, the patient is treated by different physicians, the syndrome differentiations and prescriptions can be different due to the physicians' differences of TCM theory and clinical experience. Consequently, the characteristics of TCM individualized treatment based on the individualities of the physicians can be reflected in the following two aspects (Figure 2). (1) Longitudinal direction: for the same patient, in the whole process of the disease, due to the changes of the clinical manifestations, the physician diagnoses and establishes therapeutic principle, and therapy based on his TCM theory and clinical experience at different stages. (2) Cross-sectional direction: for the same patient, at a certain stage of the disease, treated by different physicians, they may give different diagnosis, therapeutic principles and prescription according to their TCM theory basis and clinical experience.

## 3. A Pilot Clinical Study

The purpose of the study was mainly to investigate the influence of physician for individualized medicine in TBSD. And the study was conducted from April 2010 to March 2011, more detailed information about it can be known from the published dissertation [41].

### 3.1. Study Design

**Figure 3:**
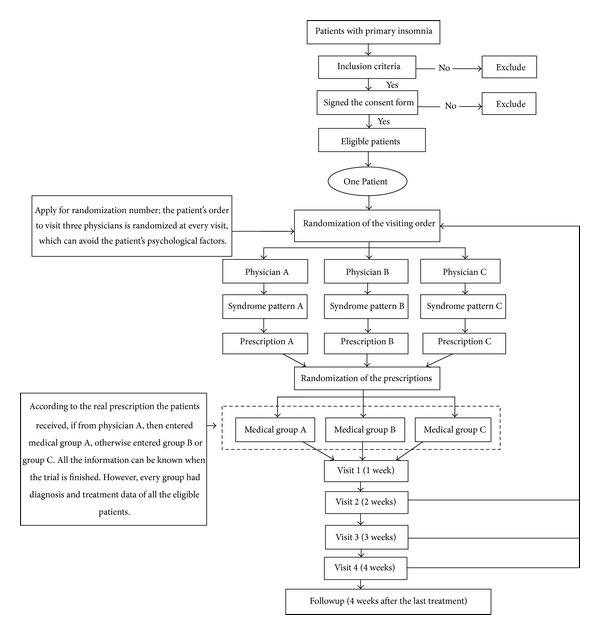
The trial design.

This was a triple-blind (with patients, physicians, and outcome assessors blinded), randomized, parallel group clinical trial. And the study was mainly about the difference of Chinese medicine physicians diagnosed and treated primary insomnia by treatment based on syndrome differentiation. The trial design was shown in Figure 3, and Table 1 summarized the timing of the trial.

### 3.2. Population and Recruitment

#### 3.2.1. Physicians

 In the study, three Chinese medicine physicians were the objects. The inclusion criteria of the physicians was as follows: (1) had senior professional titles, specialized in treating insomnia; (2) had a good effect and reputation in treating insomnia (e.g., had many patients and better appreciation feedback); (3) had unique perspectives and methods in treating insomnia (e.g., published research papers related); and (4) agreed to take part in the study. All the three physicians chosen were chief physician and experts on insomnia in Prestigious Chinese Clinician Research Laboratory of Guang'anmen Hospital, China Academy of Chinese Medical Sciences. Physician A was good at treating difficult and complicated disease, sleep disorders, and cardiopulmonary disease, in particular, the diagnosis and treatment of sleep disorders through syndrome differentiation, and regulating spleen and stomach to treat coronary heart disease. Physician B was good at treating difficult and complicated disease, in particular, the diagnosis and treatment of geriatric disease through syndrome differentiation. Physician C was mainly engaged in the sleep medical theory and clinical research.

#### 3.2.2. Patients

All patients were recruited at Guang'anmen Hospital, China Academy of Chinese Medical Sciences. The study was conducted between May and November 2010. Participants diagnosed with primary insomnia according to Chinese classification of mental disorders CCMD-3 [42] were included. The inclusion and exclusion criteria were shown in Table 2.

#### 3.2.3. Recruitment/Consent Procedures

Patients were recruited by means of poster at clinic. The patient screening was conducted by the specified physician who did not participate in the study. If a patient met the study criteria, the physician responsible for patient screening would provide him or her with written information, explaining the study in detail, and obtained written consent if he or she agreed to take part in the study. All patients' informed consent must be obtained. Any patient could not be enrolled if she/he refused or showed significant distress. Patients were free to withdraw from the study at any time.

### 3.3. Randomization and Blinding

A web central randomization system was employed in this study. Three Chinese physicians diagnosed and treated every eligible patient individually. Every Chinese physician provided syndrome patterns and the prescription according to every patient's condition. There were three prescriptions from three Chinese physicians for every patient at every visit. At the first visit, patients equally were randomized to one of the following three groups: medical group A, medical group B, or medical group C. Patients were given one of the three physicians' prescriptions. In the whole study, the randomization of group assignment only occurred at the first visit, and the randomization results were applied to the following visits. In other words, although the patients had to be diagnosed by every physician for every visit, they always received the drug prescribed by the physician who was distributed to them by randomization at the first visit. Furthermore, in order to avoid the potential bias, every patient's order to visit three physicians was randomized at every visit, meaning that the visiting order in which one patient visits the physicians may be different every time. 

In this study, the patients, physicians, and outcome assessors were blinded. The patients did not know the real prescriptions they received from which physicians and avoided their psychological factors which may influence the therapeutic effects. Meanwhile, the physicians also didn't know which patients really took their prescriptions. Moreover, at the following visits, the physicians were not allowed to ask the patients such questions like the taste or color of the Chinese herbs decoction they had taken. According to such information, the physician could guess if the patients take his prescriptions or not. In addition, the blinding implementation of the outcome assessors guaranteed the objectivity of the study. 

### 3.4. The Procedure of Study

An eligible patient who had signed informed consent stopped taking any medication before the study. In the treatment period lasting 4 weeks and including four visits, each of three physicians independently provided a syndrome pattern and prescription for every patient. Three generated prescriptions then were passed on to the pharmacy. However, whether one patient received one of three prescriptions was dependent on the result of randomization that was conducted by the authorized person using a central randomization system at the first visit. 

When a patient returned to the hospital once a week after the first treatment, a professional physician conducted an assessment on efficacy. The patient then received the secondary treatment from three Chinese physicians, repeating the same procedure as the first one. The whole study lasted 8 weeks, including 4-week treatment and followup happening at the 4th week after the last treatment.

In order to minimize potential bias and to keep the blind condition of study as possible as one can, all researchers participating in the study were divided into three groups: clinical study group, diagnosis and assessment group, and quality control and statistical analysis group. The study was approved by the Institutional Review Board of the Guang'anmen Hospital, China Academy of Chinese Medical Sciences.

### 3.5. Interventions

After randomization, the three-group patients were to receive one of three Chinese physicians' prescriptions. Every Chinese physician diagnosed the patients and prescribed individually based on patients' syndrome differentiation and their own clinical experience. During the whole treatment, the physicians could make necessary adjustment in their medicinal prescription depending on the condition of the disease. The prescriptions of the physicians were pure Chinese herbs. Patients of the three groups were required to take 150 mL (50°C) twice daily for 4 weeks. For the duration of the trial, the patients were not allowed to take any concomitant medications associated with the treatment of insomnia. 

### 3.6. Outcome

#### 3.6.1. Primary Efficacy Indicator

In this study, the primary outcome was the Pittsburgh Sleep Quality Index (PSQI). It was a self-rated questionnaire used to assess sleep quality and disturbances over a 1-month time interval. Nineteen individual items generated seven component scores: subjective sleep quality, sleep latency, sleep duration, habitual sleep efficiency, sleep disturbances, use of sleeping medication, and daytime dysfunction. Many studies had demonstrated that the PSQI was a sensitive, reliable, and valid tool to assess the quality of sleep [43–45]. The evaluation standard for clinical efficacy of PSQI was as follows: (1) clinical cure: at the last visit, the reduction score rate of PSQI ≥80%, comparing with the baseline; (2) markedly effective: at the last visit, the reduction score rate of PSQI ≥50%, comparing with the baseline; (3) effective: at the last visit, the reduction score rate of PSQI ≥30%, comparing with the baseline; and (4) ineffective: at the last visit, the reduction score rate of PSQI <30%, comparing with the baseline.

#### 3.6.2. Secondary Efficacy Indicators

The secondary efficacy indicators included the primary symptoms scores of insomnia and symptom scale of Chinese medicine. The primary symptoms of insomnia were as follows: difficulty initiating sleep, often nocturnal awakenings, difficulty maintaining sleep or not easy to sleep again after waking up, sleep not deep, too much dream, wake up early in the morning, uncomfortable after waking up, fatigue, and daytime sleepiness. Each symptom with four scoring options (absent = 0, mild = 2, moderate = 4, or severe = 6), while the symptoms scale of Chinese medicine included headache, dizziness, spontaneous sweating, night sweating, dreaminess, amnesia, anorexia, lassitude of spirit and lack of strength, oppression in the chest, daytime sleepiness, irritability, palpitations, epigastric fullness, tinnitus, thirst, bitter taste in the mouth, abundant sputum, yellow urine, dry stool, and sloppy stool. Each item with four scoring options (absent = 0, mild = 1, moderate = 2, or severe = 3). The clinical efficacy standard of symptoms scores of insomnia and symptom scale of Chinese medicine were as follows: (1) clinical cure: at the last visit, TCM symptoms scores reduction rate ≥95%, comparing with the baseline; (2) markedly effective: at the last visit, TCM symptoms scores reduction rate ≥70%, comparing with the baseline; (3) effective: at the last visit, TCM symptoms scores reduction rate ≥30% comparing with the baseline; and (4) ineffective: at the last visit, TCM symptoms scores reduction rate <30%, comparing with the baseline. 

The total efficacy rate was calculated using the following formula: clinical cure rate + markedly effective rate + effective rate. The pulse condition and tongue manifestation were described in words. In every visit the specified physician who didn't participate in the study took notes about such information. 

### 3.7. Safety Assessment

During the trial, adverse events and vital signs were observed in detail and documented using the case report forms at every visit. The major indicators for vital signs include breath, temperature, systolic blood pressure, and diastolic pressure, pulse. Generally, any unexpected symptom, vital sign or sickness, as long as they caused discomfort, should be recorded as an adverse event. The starting date, the ending date, the degree, the relations with the trial medicine, and whether they dropped out of the study should be recorded correspondingly. If necessary, the patients would receive relevant treatment. If the adverse event still existed, the followup should go on until the adverse event disappeared.

### 3.8. Statistical Analysis

#### 3.8.1. Sample Size

The study was supposed to evaluate the influence of physician to personalized medicine in the process of TBSD, which was based on primary insomnia among three prestigious physicians by observing their daily clinical practices. However, it was very difficult to estimate the sample size because of lack of a previous relevant study. Considered the limitations of time and research efforts, 33 patients were recruited (3 withdrew). Every patient received diagnosis and treatment by three physicians at every visit, that is, the whole sample size reached 90.

#### 3.8.2. Data Collection

A highly structured electronic medical records system was employed to collect and save the clinical data in this study. And it was based on the standardized and normalized clinical terminology.

#### 3.8.3. Analysis Methods

The Statistical Package for the Social Sciences 17.0 was used for the statistical analysis. The mean and standard deviation were applied to the continuous variables and percentages to the categorical variables. For comparison of two independent samples, the *t* test and analysis of variance were applied for continuous variables and the Chi-square test for categorical variables. The therapeutic effect analysis was conducted according to the case number that received real prescription. For the difference analysis of TBSD among the three physicians, the whole patients were taken into account, because every patient received diagnosis and treatment by three physicians at every visit.

## 4. Results 

### 4.1. Study Population

A total of 33 patients were recruited between May and November 2010; 3 patients withdrew from the trial because they were not able to go to see the doctors on time for business trip. The average age of the patients is 51.63 ± 6.33 years. There were 19 female patients (63.3%). Among these 30 patients, 9 were randomized into medical group A, 11 into the medical group B, and 10 into the medical group C. No obvious adverse reaction was observed in the three groups.

### 4.2. The Therapeutic Effect of PSQI and the TCM Symptoms Measuring Scale

The PSQI score of the three medical groups based on 4 weeks' treatments was shown in Table 3. And the clinical therapeutic effect rate based on the PSQI and the TCM symptoms measuring scale were presented in Table 4. The result of PSQI showed that the overall clinical effect rate of the three physicians was 66.67%, medical group A was 66.67%, medical group B was 72.73%, and medical group C was 60%, respectively. The clinical effect rate based on the TCM symptoms measuring scale in medical group A was 66.66%, medical group B was 81.82%, and medical group C was 90%, respectively. For the clinical efficacy comparison among the three physicians, however, there was no significant difference. The results maybe suggested that physician B was good at improving the sleep disorders, but physician C was good at improving the other symptoms and signs except the sleep disorders.

### 4.3. The Syndrome Differentiation Analysis of the Three Chinese Physicians

#### 4.3.1. The Overall Syndrome Differentiation Conditions

The top 10 syndrome differentiations of the primary insomnia used by the three physicians are four areas heat, phlegm, blood and liver. Heat disturbing heart-mind, Phlegm-heat, disharmony between heart and kidney, blood stasis, blood deficiency, and liver stagnation were the common syndrome patterns, which were consistent with the syndrome differentiation characteristics of each physician. The results were illustrated in Table 5.

#### 4.3.2. The Respective Syndrome Differentiation Condition of the Three Physicians

As shown in Table 6, the results of syndrome differentiation of the three physicians were different. The syndrome differentiation of blood deficiency and liver stagnation were the main patterns for physician A. For physician B, the syndrome differentiation of heat disturbing heart-mind and phlegm-heat were the main pathogenesis of insomnia. The opinion of physician C mainly focused on the “blood;” blood stasis and blood deficiency were the entry points for him to treat insomnia. 

#### 4.3.3. The Differences of Diagnosis Based on Syndrome Differentiation among the Three Physicians at the First Treatment

Table 7 showed the differences of diagnosis based on syndrome differentiation among the three physicians at the first treatment. All the three physicians conducted four diagnostic information acquisition and prescription based on the syndrome differentiation. It is shown obviously that the results of syndrome differentiation were different. For patient number 6, the syndrome differentiation of physicians A was kidney deficiency, while physicians B was blood stasis and blood not nourish heart and disturbing heart-mind, the diagnosis of physicians C was stagnation of qi and blood stasis. As shown in Table 7, the syndrome differentiations of other patients by the three physicians were quite different.

#### 4.3.4. The Syndrome Differentiation of the Three Physicians for One Patient in the Whole Treatment Period

In order to reflect how different the three physicians treating the same patient, we randomly selected one patient and analyzed the changes of chief complaint, history of present illness, clinical information collected, syndrome patterns, the treating principle and prescriptions at the different visits. In Table 8, the results showed that the clinical information reflecting the feature of insomnia such as the initiating sleep time, the quality of sleep, were more or less the same. However, there existed differences in the description and judgment of the tongue and pulse manifestation, as well as the other symptoms and signs. Correspondingly, the syndrome pattern, treating principle, and prescriptions were much different. In the whole process of the treatment, every physician diagnosed and established therapeutic principle, and prescription differently at four visits for the same patient. Meanwhile, at the same visit, they gave different syndrome pattern diagnosis and therapeutic principle for the same patient. 

## 5. Discussion

As the main clinical practice method, the treatment based on syndrome differentiation is a complex and flexible process where the physician and patient participate in the treatment together. This paper provides our perspective on the TBSD that it is an interactive process of the subject (physician) and the object (patient), while the physician plays an important role for individualized medicine. The results show that the clinical observations, diagnoses, and herb prescriptions have significant differences among 3 physicians. This confirms the influence of physician in different stages of TBSD. Accordingly, the evaluation methods for therapeutic effect of TBSD should take this interactive process as a whole. it conforms to the real world clinical research paradigm [46] of TCM, which reflects the characteristics of treatment based on syndrome differentiation, such as individualized treatment, dynamic, and complex interventions.

Recent studies or theories have emphasized the individualities of the patients, in particular, the constitution of the patients are more concerned [47–49]. The Chinese constitutional theory also provides methods and tools for individualized treatment [50]. Moreover, the methods of systems biology [51–55] are applied to promote the classification of TCM constitution types and syndrome patterns, as well as explain the individual differences in response to treatment effect and the differences in adverse drug reactions. However, in our opinion, the generation of TCM individualized treatment not only depends on the patient's individualities, but also depends more on the individualities of the physicians. 

Usually, the learning, experience and the habit of diagnosis and treatment form the individualities of a physician [40] particularly the TCM theory they are applying and their clinical experience. The physicians' leading role in the interactive process is thus presented in the following aspects: first, the conception and formation process of TCM syndrome highlights the important thinking process of the physicians; second, the cause analysis of the TCM individualized diagnosis and treatment was embodied by the different TCM theory schools and the clinical experience of the physicians; and third, the characteristics analysis of the TCM individualized diagnosis and treatment based on the individualities of the physicians was embodied in the cross-sectional and longitudinal direction.

Furthermore, the randomized, triple-blind trial we conducted fully showed the whole process of TBSD of the three physicians. The individualities of the physicians were reflected in the syndrome diagnosis as well as the treating principle and prescription. For the primary insomnia patients, they received the TBSD by the three physicians individually and independently; the treatment was comparably effective. Nevertheless, the physicians' reasoning of syndrome differentiation was different. Physician A particularly emphasized blood deficiency and liver stagnation, while physician B considered that the heat disturbing heart-mind and phlegm-heat were the main pathogenesis of insomnia, and physician C mainly focused in the “blood,” that is, blood stasis and blood deficiency were the entry point for him to treat insomnia. Therefore, at the first visit, the syndrome differentiation results of every patient among the three physicians were quite different. In addition, for a given patient, the different emphasis of clinical information, syndrome differentiation, as well as the treating principle and prescriptions of the physicians, were also obvious in the cross-sectional direction and longitudinal direction.

At present, the N-of-1 trials are gradually applied in TCM, for instance, to evaluate the dose-effect relationship of Niuhuang-Jiangya capsule in the treatment of mild to moderate essential hypertension [56], the treatment based on syndrome differentiation of chronic kidney disease [57, 58], after chemotherapy bone marrow suppression [59] and hypertensive intracerebral hemorrhage [60]. Usually N-of-1 trials are multiple-cycle, double blind, placebo, or positive drug controlled crossover trial using standardized measures of effect. And they are mainly used for testing the effectiveness of medicines in individual patients [61]. Generally, the diseases are defined as nonself-limiting chronic diseases with more stable condition and long-term medication in N-of-1 trials [62, 63]. While the study we designed is to explore the common or individual empirical regularities of different physicians in the process of treatment based on syndrome differentiation, and the process is considered as a whole. Consequently, compared with the N-of-1 trials, there are no wash-out periods during the treatment in our study; the intervention is not standardized or fix prescription; the disease is not limited to the chronic illnesses. Furthermore, the study design keeps all the characteristics of treatment based on syndrome differentiation and takes the interactive process of the physician and patient as a whole. While in the N-of-1 trials, the interactive process is separated. 

The influence of physicians for individualized medicine may also provide ideas and cues for clinical research in TCM field. Especially the experience inheritance research of the prestigious physicians and the clinical research involving TCM clinicians. That is, physician should be considered as a main factor to TBSD related clinical research.

Of course, it is still in the exploratory stage, there are some limitations. This study used a short treatment period and followup and a relatively small number of patients; the outcome measures of primary insomnia could choose more standardized and scientifically validated indicators except PSQI, such as the Spiegel sleep questionnaire [64], the sleep dysfunction rating scale [65], the total sleep time, and the sleeping rate. Moreover, there are difficulties in the future spread and application in multiple centers. Because the three physicians in this study are all in the same center, they may diagnose and treat the patient at the same visit; accordingly, the application is feasible. Therefore, the administration mode of the study needs to change if it refers to multiple centers. We assume that the physicians of the same TCM theory school can be divided into the same group, and then each of them can join the study flexibly. 

So, there is ample room to enhance the evaluation of efficacy and sum up the common or individual regularity of the physicians by further studies. Recently, we have modified the study design, added the placebo control group, and applied it in exploring effective core drug patterns in primary insomnia [66].

## 6. Conclusions

TBSD is considered as an interactive process of the subject (physician) and the object (patient), in which the influence of the physician is an important factor in clinical observation, diagnosis, and treatment. This means that physician plays important role in individualized medicine. The results of this study confirm that although, there exists significant differences in syndrome differentiation, the treating principles, and the prescriptions of the approaches used by 3 physicians achieve with comparable performance. 

## Figures and Tables

**Table 1:** Calendar summary.

Period	Screening	Treatment				Follow-up
Week (W)	0 W	1st W	2nd W	3rd W	4th W	5th–8th week after the end of treatment
Inclusion and exclusion criteria	×					
Informed consent	×					
Demography	×					
Past medical history and treatment history	×					
Self-rating anxiety scale	×					
Beck depression inventory	×					
Pittsburgh sleep quality index	×	×	×	×	×	×
TCM symptoms and signs	×	×	×	×	×	×
Prescription (include every herb and dose)		×	×	×	×	
Vital signs	×	×	×	×	×	
Adverse event		×	×	×	×	

**Table 2:** Inclusion and exclusion criteria.

Inclusion criteria	
(1) Patients who met the diagnosis standard of primary insomnia, with Spiegel scale >18.	
(2) Patients aged 18 to 65 years.	
(3) Signed the informed consent.	

Exclusion criteria	
(1) Total sleep time <2 hours.	
(2) Secondary insomnia caused by or comorbid with physical illness and mental disorders and so forth.	
(3) Patients with severe anxiety and depression, self-rating anxiety scale score >60, and beck depression inventory score <8.	
(4) Patients who have disease complicated with hypertension, diabetes, stroke, and coronary heart disease.	
(5) Patients with history of alcoholic or drug abuse.	
(6) Pregnant or preparing for pregnancy or lactating women.	
(7) Patients who are involved in other trials at the same time.	
(8) Patients who have allergic constitution or known to be allergic to the Chinese herbs.	
(9) Patients with poor compliance or other reasons that the investigator considered not to be appropriate to participate in this trial.	

**Table 3:** The analysis of reducing score rate of PSQI.

Random number	Total PSQI score at the 1st visit	Total PSQI score at the 5th visit	Difference between the 5th and 1st visit	Reducing score rate of PSQI	Medical group
1	16	3	13	81.25%	C
3	9	5	4	44.44%	B
4	20	11	9	45.00%	B
5	13	12	1	7.69%	C
6	9	6	3	33.33%	A
7	15	4	11	73.33%	A
8	13	7	6	46.15%	C
9	11	6	5	45.45%	B
10	13	13	0	0.00%	B
13	13	12	1	7.69%	C
14	14	12	2	14.28%	B
15	14	9	5	35.71%	A
16	16	8	8	50.00%	B
17	13	11	2	15.38%	A
18	12	4	8	66.67%	C
19	10	1	9	90.00%	B
20	13	12	1	7.69%	C
21	12	6	6	50.00%	A
22	8	2	6	75.00%	C
23	12	9	3	25.00%	A
24	18	4	14	77.78%	B
25	16	8	8	50.00%	A
26	13	5	8	61.54%	B
27	13	4	9	69.23%	C
28	11	2	9	81.82%	C
29	13	7	6	46.15%	A
30	17	12	5	29.41%	B
31	16	12	4	25.00%	A
32	16	11	5	31.25%	B
33	13	10	3	23.08%	C

**Table 4:** The clinical effect rate based on the PSQI score and TCM symptoms measuring-scale.

	Medical group A		Medical group B		Medical group C	
	PSQI score	TCM symptoms measuring-scale	PSQI score	TCM symptoms measuring-scale	PSQI score	TCM symptoms measuring scale
Clinical efficacy rate	66.67%	66.66%	72.73%	81.82%	60%	90%
Clinical cure	0%	0%	9.09%	0%	20%	0%
Markedly effective	33.33%	33.33%	27.27%	36.36%	30.00%	20%
Effective	33.33%	33.33%	36.36%	54.55%	10.00%	70%
Ineffective	33.33%	33.33%	27.27%	18.18%	40.00%	10%

*P* > 0.05, the comparison between the three groups.

**Table 5:** The frequency of overall syndrome differentiation condition.

Syndrome pattern name	Syndrome pattern frequency*
Heat disturbing heart-mind	107
Phlegm-heat	64
Disharmony between heart and kidney	41
Blood stasis	36
Blood deficiency	32
Liver stagnation	30
Liver heat	24
Exuberance of liver	21
Blood not nourishing heart	21
Qi deficiency of heart and gallbladder	19

*The syndrome pattern frequency ≥ 10.

**Table 6:** The syndrome differentiation of the three physicians.

Physicians	Syndrome pattern name	Case number	Frequency*
A	Blood deficiency	12	31
	Liver stagnation	10	23
	Exuberance of liver	8	21
	Kidney deficiency	7	18
	Liver heat	7	15
	Stomach heat	6	15
	Heat disturbing heart-mind	7	14
	Phlegm-heat	7	13

B	Heat disturbing heart-mind	29	93
	Phlegm-heat	15	50
	Disharmony between heart and kidney	9	29
	Blood not nourish heart	7	21
	Endogenous heat due to Yin deficiency	7	16
	Blood stasis	5	14
	Endogenous heat	3	10

C	Blood stasis	13	22
	Liver blood deficiency	9	16
	Heart blood deficiency	8	15
	Qi deficiency of heart and gallbladder	7	15
	Phlegm-heat disturbance	6	15
	Blood deficiency of liver and heart	5	12
	Disharmony between heart and kidney	7	12
	Fever due to deficiency	5	10
	Yin deficiency	5	10

*The syndrome pattern frequency ≥ 10.

**Table 7:** The syndrome differentiation of the three physicians at the first treatment.

Random number	Physician A	Physician B	Physician C
06	Kidney deficiency	(i) Blood stasis (ii) Blood not nourish heart and disturbing heart-mind	(i) Stagnation of qi and blood stasis (ii) Dampness
21	(i) Kidney deficiency (ii) Exuberance of liver (iii) Dampness blockage	Phlegm-heat	Blood stasis
23	(i) Kidney deficiency (ii) Exuberance of liver (iii) Dampness due to the deficiency of spleen	(i) Disharmony between heart and kidney (ii) Heat disturbing heart-mind	(i) Discomfort in stomach and spleen (ii) Qi deficiency of heart and gallbladder
25	Discomfort in stomach	(i) Endogenous heat due to Yin deficiency (ii) Heat disturbing heart-mind	(i) Kidney deficiency (ii) Exuberance of liver (iii) Disharmony between heart and kidney
15	(i) Qi and blood deficiency (ii) Malnutrition of heart-mind	(i) Endogenous heat due to Yin deficiency (ii) Heat disturbing heart-mind	Fever due to deficiency
17	(i) Qi and blood deficiency (ii) Endogenous heat due to Yin deficiency (iii) Heat disturbing heart-mind	Blood not nourish heart	(i) Yin deficiency of liver and kidney (ii) Disharmony between heart and kidney
29	(i) Kidney deficiency (ii) Liver heat (iii) Stomach stagnation	Phlegm-heat disturbing heart-mind	(i) Phlegm-heat disturbing heart mind (ii) Dampness-heat
01	Blood deficiency and liver stagnation	(i) Phlegm-damp transform into heat (ii) Heat disturbing heart-mind	Liver stagnation and deficiency of spleen
05	Phlegm-heat disturbing heart-mind	Phlegm-heat disturbing heart-mind	Yin deficiency of heart
13	(i) Yin deficiency (ii) Liver and stomach depression transforming into heat	Phlegm-heat disturbing heart-mind	(i) Blood deficiency of liver and heart (ii) Hyperactivity of fire due to yin deficiency
08	Kidney deficiency	(i) Disharmony between heart and kidney (ii) Phlegm-heat disturbing heart-mind	(i) Qi deficiency of heart and gallbladder (ii) Qi stagnation hurt heart-mind
18	Phlegm-heat	Phlegm-heat disturbing heart-mind	Yin deficiency of liver and Kidney
27	(i) Kidney deficiency (ii) Exuberance of liver	(i) Blood stasis (ii) Heat disturbing heart-mind	(i) Phlegm-fire disturbing heart (ii) Dampness-heat diffused downward
28	(i) Liver stagnation (ii) Blood deficiency	(i) Blood stasis (ii) Blood not nourishing heart	Liver stagnation and deficiency of spleen
33	(i) Liver stagnation (ii) Blood deficiency (iii) Syndrome of fu-viscera of Yang ming	(i) Blood stasis (ii) Heat disturbing heart-mind	Stagnation of qi and blood stasis
20	(i) Blood deficiency (ii) Liver stagnation (iii) Phlegm disturbance	(i) Disharmony between heart and kidney (ii) Heat disturbing heart-mind	Blood deficiency of liver and heart
03	Blood deficiency and liver stagnation	(i) Endogenous heat due to Yin deficiency (ii) Heat disturbing heart-mind	Blood stasis
04	Stagnant heat of liver and stomach	(i) Disharmony between heart and kidney (ii) Phlegm-heat disturbing heart	Liver fire flaring up
10	Kidney deficiency and liver stagnation	(i) Disharmony between heart and kidney (ii) Blood not nourishing heart	Yin deficiency of liver and kidney
14	(i) Qi and blood deficiency (ii) Stagnant heat of liver and stomach	(i) Blood not nourishing heart (ii) Endogenous heat due to Yin deficiency	Blood stasis
16	(i) Blood deficiency (ii) Exuberance of liver (iii) Phlegm blockage	(i) Disharmony between heart and kidney (ii) Heat disturbing heart-mind	Dampness-heat diffused downward
19	(i) Qi deficiency of heart and gallbladder (ii) Phlegm blockage	Phlegm-heat disturbing heart-mind	(i) Qi deficiency of heart and gallbladder (ii) Endogenous heat due to gallbladder heat (iii) Blood stasis
26	(i) Qi and blood deficiency (ii) Exuberance of liver disturbing heart-mind	(i) Disharmony between heart and kidney (ii) Endogenous heat due to Yin deficiency	Blood stasis
30	(i) Liver stagnation and deficiency of spleen (ii) Exuberance of liver	(i) Blood not nourishing heart (ii) Endogenous heat	Blood deficiency of liver and heart
32	(i) Kidney deficiency (ii) Exuberance of liver	Phlegm-heat	Phlegm-heat
24	(i) Blood deficiency (ii) Exuberance of liver	(i) Blood stasis (ii) Phlegm-heat disturbing heart-mind	Liver stagnation and deficiency of spleen

**Table 8:** The syndrome differentiation of the three physicians for one patient in the treatment period.

		Physician A	Physician B	Physician C
The 1st treatment	Chief complaint and history of present illness	*The primary symptoms associated with insomnia* (i) Insomnia for more than 20 years (ii) Sometimes difficulty initiating sleep (iii) Easy to wake up at night (iv) Not too much dream than before *Other symptoms accompanied* (i) Forgetfulness (ii) Dizziness (iii) Nausea and want to vomit (iv) Upper abdominal fullness (v) Impatience but controllable (vi) Good appetite (vii) Frequent stool (viii) Chronic atrophic gastritis (ix) Took Chinese medicine herbs before	*The primary symptoms associated with insomnia* (i) Insomnia for more than 20 years (ii) Difficulty initiating sleep (iii) Easy to wake up in 1-2 hours (iv) Not easy to sleep again after waking up *Other symptoms accompanied* (i) forgetfulness (ii) Sometimes dizziness (iii) A few months ago attacked with nausea and want to vomit (iv) Frequent stool, 3 or 4 times per day (v) To be bloated in head	*The primary symptoms associated with insomnia* (i) Insomnia for more than 20 years (ii) Go to bed at 22:00 every night (iii) Initiate sleep in 1-2 hours (iv) One or two days difficulty initiating sleep in a week (v) Not too much dream than before (vi) Wake up at 4:00 or 5:00 in the morning *Other symptoms accompanied* (i) To be bloated in top head (ii) Memory loss (iii) No palpitation (iv) No shortness of breath (v) The mood is ok.
	Signs	(i) Dark complexion with chloasma (ii) Tongue: trembling, teeth-printed, a little deep of the tongue quality (iii) Pulse: stringy and thready pulse	(i) Tongue: deep red tongue quality, teeth-printed, thin and yellow fur (ii) Pulse: stringy, thready, and slow pulse	(i) Dark spots on the face, look dull, bulbar conjunctiva without congestive (ii) Tongue: pale of the tongue quality, thin and white fur (iii) Pulse: stringy and slow pulse
	Syndrome pattern	Main pattern: blood deficiency	(i) Main pattern: blood not nourish heart (ii) Secondary pattern: blood stasis	(i) Main pattern: stagnation of liver Qi and spleen deficiency (ii) Secondary pattern: blood stasis
	Treating principle and prescription	(i) Principle: nourishing blood for tranquillization (ii) Prescription: Gui Pi decoction	(i) Principle: nourishing and promoting blood for tranquillization (ii) Prescription: TianWang Bu Xin Dan and Si Wu decoction	(i) Principle: dispersing stagnated liver Qi for relieving Qi stagnation (ii) Prescription: Jie Yu San Jie decoction

The 2nd treatment	Chief complaint and history of present illness	*The primary symptoms associated with insomnia* (i) Sometimes the initiate sleep is improved (ii) Wake up per hour (iii) From midnight to 4:00 in the morning cannot sleep *Other symptoms accompanied* (i) Dizziness (ii) No nausea (iii) Upper abdominal fullness (iv) Dampness in the ears (v) Tongue ache for a whole night (vi) Scurrying pain of the body (vii) Stool irregular and shapeless (viii) Bitter taste in mouth and thirst (ix) Occasionally urticaria attacks	*The primary symptoms associated with insomnia* (i) The initiate sleep sometimes is rapid sometimes is slow (ii) Easy to wake up during the sleep *Other symptoms accompanied* (i) Dizziness (ii) Bitter taste and dry mouth (iii) Stool 2 times per day (iv) Head discomfort	*The primary symptoms associated with insomnia* (i) The quality of sleep is not so ideal (ii) Difficulty initiating sleep (iii) Not easy to sleep again after waking up in the night (iv) Too much dream (v) Get up in 5:00 in the morning *Other symptoms accompanied* (i) The urine and stool are normal (ii) Few sweating in the sleep (iii) Bitter taste and dry mouth (iv) There are rashes recently
	Signs	(i) Tongue: the tip of tongue is red, yellow fur, teeth-printed (ii) Pulse: stringy and thready pulse	(i) Tongue: red tongue quality, thin and yellow greasy fur (ii) Pulse: stringy and thread pulse	(i) There are a few pigmentation, no rashes (ii) Tongue: red tongue quality, thin yellow fur and teeth-printed (iii) Pulse: deep and thready pulse
	Syndrome pattern	Liver heat and phlegm blocking	(i) Main pattern: blood not nourishing heart (ii) Secondary pattern: phlegm-heat	Main pattern: souls and spirits are uneasy
	Treating principle and prescription	(i) Principle: clear liver heat and remove phlegm (ii) Prescription: Huang Lian Wen Dan decoction	(i) Principle: nourishing blood for tranquillization and removing phlegm-heat (ii) Prescription: regulation based on TianWang Bu Xin Dan and Wen Dan decoction	(i) Principle: comfort the souls and brain for tranquillization (ii) Prescription: Zi Ni Fang (the formula prescribed according to the experience of physician C)

The 3rd treatment	Chief complaint and history of present illness	*The primary symptoms associated with insomnia* sleep for 2-3 hours *Other symptoms accompanied* (i) The tongue ache disappears (ii) Aphtha (iii) Scurrying pain of the body (iv) The urticaria attacks (v) Upper abdominal fullness is relieved (vi) Fear of cold (vii) Dizziness to close the eyes (viii) Bitter taste and dry mouth (ix) Thirsty (x) Stool irregular and shapeless is remission	*The primary symptoms associated with insomnia* (i) The initiate sleep is slow (ii) Often awake the whole night *Other symptoms accompanied* (i) Stomach bloating is remission (ii) Fear of cold	*The primary symptoms associated with insomnia* (i) Easy to wake up in the night (ii) Sometimes difficulty initiating sleep (iii) Too much dream *Other symptoms accompanied* (i) Sweating in the sleep (ii) Bitter taste and dry mouth (iii) Normal diet (iv) The stool and urine are normal (v) No chest tightness (vi) No shortness of breath (vii) Rash itching at night
	Signs	(i) Yellow complexion, not shiny (ii) Tongue: deep red tongue quality, yellow fur (iv) Pulse: stringy and thready pulse	(i) Tongue: red tongue quality, thin yellow greasy fur and teeth-printed on the edge (ii) Pulse: thready and stringy pulse	(i) A few pigmentation on the face (ii) No rashes (iii) Sonorous voice (iv) Tongue: pale tongue quality, white fur (v) Pulse: deep and thready pulse
	Syndrome pattern	Main pattern: liver heat and phlegm blocking	Main pattern: phlegm-heat disturbing heart mind	(i) Main pattern: syndrome of phlegm-heat attacking internally (ii) Secondary pattern: blood stasis
	Treating principle and prescription	(i) Principle: clear liver heat and remove phlegm (ii) Prescription: regulation based on Huang Lian Wen Dan decoction	(i) Principle: remove phlegm and clear heart heat (ii) Prescription: regulation based on Huang Lian Wen Dan decoction	(i) Principle: clear heat for removing phlegm (ii) Prescription: regulation based on Shi Wei Wen Dan decoction

The 4th treatment	Chief complaint and history of present illness	*The primary symptoms associated with insomnia* (i) Initiate sleep for 1-2 hours (ii) Easy to wake up *Other symptoms accompanied* (i) The eyes are too dry to close (ii) The aphtha is healed (iii) The urticaria attacks in the night (iv) Legs and back ache (v) Upper abdominal fullness is relieved belching (vi) Fear of cold (vii) Dizziness to close the eyes (viii) Dizziness (ix) No bitter taste in the mouth but dry (x) Thirsty (xi) No falling feeling of the abdomen (xii) The stool is regular	*The primary symptoms associated with insomnia* (i) The initiate sleep is slow (ii) Easy to wake up *Other symptoms accompanied* (i) Stomach bloating is remission (ii) Occasionally belching (ii) Sometimes the urticaria attacks in night	*The primary symptoms associated with insomnia* (i) The initiate sleep is normal (ii) Easy to wake up in the night (iii) Not easy to sleep again after waking up (iv) Occasionally feel sleepy during the daytime *Other symptoms accompanied* (i) Not much night urine (ii) No sweating (iii) No upset (iv) No palpitations (v) Often sigh (vi) No headache (vii) No dizziness (viii) Menopause
	Signs	(i) Tongue: red tongue quality of the tip, yellow fur and teeth-printed on the edge (ii) Pulse: stringy pulse	(i) Tongue: red tongue quality, yellow greasy fur (ii) Pulse: thready and slow pulse	(i) Tongue: red tongue quality, white fur (ii) Pulse: deep and slow pulse
	Syndrome pattern	Main pattern: disharmony of Yin and Yang	Main pattern: phlegm-heat disturbing heart mind	Main pattern: syndrome of deficiency of heart yin
	Treating principle and prescription	(i) Principle: Harmony Yin and Yang (ii) Prescription: Gui Zhi add Long Gu and Mu Li decoction	(i) Principle: remove phlegm and clear heart heat (ii) Prescription: regulation based on Huang Lian Wen Dan decoction	(i) Principle: nourishing heart for tranquillization (ii) Prescription: regulation based on Jing Xin decoction
